# Conservative Surgical Management of Stage I Bisphosphonate-Related Osteonecrosis of the Jaw

**DOI:** 10.1155/2014/107690

**Published:** 2014-02-06

**Authors:** Paolo Vescovi, Elisabetta Merigo, Marco Meleti, Maddalena Manfredi, Carlo Fornaini, Samir Nammour, Giovanni Mergoni, Amin Sarraj, Jose V. Bagan

**Affiliations:** ^1^Unit of Oral Pathology and Laser-Assisted Oral Surgery, Department of Biomedical, Biotechnological and Translational Sciences, University of Parma, 43100 Parma, Italy; ^2^Université de Liège, 4000 Liège, Belgium; ^3^University of Valencia, 46010 Valencia, Spain

## Abstract

*Purpose*. To report the efficacy of conservative surgical treatment for stage I bisphosphonate-related osteonecrosis of the jaw (BRONJ). *Materials and Methods*. This study reports the clinical outcomes of 63 patients treated for BRONJ stage I (according to Ruggiero's staging system) at the Oral Pathology and Laser-Assisted Surgery Unit of the University of Parma between January 2004 and January 2011. Surgical interventions were performed, under local analgesia, in patients unresponsive for a period of six months to noninvasive treatments such as cycles of local or systemic antibacterial therapy combined or not to low level laser therapy, ozone therapy, or Hyperbaric Oxygen Therapy. All interventions were performed after the consultation of oncologist or physician. *Results*. In our experience, conservative surgical treatment is associated with the highest number of BRONJ healed sites in stage I disease. Complete healing was observed in 92.6% of sites surgically treated. *Conclusions*. This study confirms that treatment of patients affected by minimal bone exposition, (stage I of BRONJ), through conservative surgical strategies, possibly with laser, may result in a high control of the disease in the long term.

## 1. Introduction

Bisphosphonate-related osteonecrosis of the jaw (BRONJ) is currently defined as an area of exposed bone in the maxillofacial region that has persisted for more than 8 weeks in a patient on previous or current treatment with a bisphosphonate and without history of radiation therapy to the jaws. Despite this definition, many cases of nonexposed variant of BRONJ have been reported.

Many pathogenetic hypotheses have been put forward but none of them could explain the peculiar character of this disorder. Osteoclasts are the main target of bisphosphonates, with the suppression of osteoclast-mediated bone remodelling. Because remodelling is high in the jaw, remodelling suppression hypothesis has been firstly proposed [1].

Even if BRONJ seems to be a primarily bone condition, some studies showed a toxic effect of bisphosphonates (BP) on the oral epithelium with inhibition of normal soft tissue healing. Because epithelialisation is an essential step in postintervention wound healing, it has been hypothesized that the soft tissue of the oral mucosa could be a key factor in BRONJ development. Moreover, a relevant role has been advocate for the antiangiogenetic effect of BP, particularly for the possible failure of healing processes with exposure of bone, which could then become necrotic. Other factors likely involved in the BRONJ etiopathogenesis are the anatomic site, bacterial infection, diabetes, smoking, concurrent medications, and genetic predisposition [1].

BRONJ is a multifactorial disease and it is therefore difficult to develop an aetiological therapy.

BRONJ management is controversial: there are no evidence-based guidelines in the literature associated with good results for a long-term followup, in particular regarding surgical procedures [2]. The main purposes of each treatment are to reduce pain and infection and slow the progression of the disease. Most of the authors privilege a noninvasive approach especially for asymptomatic stages of BRONJ (stage I in Ruggiero'staging system) (Table 1) [3].

Temporary suspension of BPs offers no short-term benefit, whilst long-term discontinuation may be beneficial in stabilizing sites of ONJ and reducing clinical symptoms [3].

The position paper of AAOMS suggested the use of oral antimicrobial rinses for stage I and systemic antibiotic therapy (penicillin, metronidazole, quinolones, clindamycin, doxycycline, and erythromycin) for symptomatic stages (stages 0, II, and III) (Table 1).

The main problem of local or systemic antibacterial therapy is the shortness of clinical results producing improvement of abscess, pain, and swelling which are usually followed by a relapse of infection and symptoms after an average of three weeks. Another aspect is that these patients are usually old and under chemotherapy, are debilitated by malignancies, and are thus not able to bear the side effects of prolonged (and sometimes permanent) antibiotic schedules. Furthermore, the evolution of disease and the uncontrollable transition from stage I to advanced stages of BRONJ are not unlikely [4].

Recently, Teriparatide (N-terminal 34 amino acids of recombinant human parathyroid hormone) was reported for medical treatment of BRONJ [5]. This compound increases bone density stimulating osteoblastic bone formation and as well as bone remodelling [6]. However the treatment with such a drug should be limited to 2 years because preclinical studies showed increased risk of osteosarcoma for long-term exposure. For this reason Teriparatide should not be recommended for patients with metastatic cancer [5, 6].

Pentoxifylline and *α*-tocopherol in addition to antimicrobial therapy induced a 74% decrease in area of bone exposure and symptoms in BRONJ patients also in early stages of disease [7].

*In vitro* studies support the hypothesis that local or systemic treatment with Geranylgeraniol (GGOH) improves viability and migration capacity of osteoblasts, fibroblasts, and endothelial cells with possible mucosal healing also in stage I of BRONJ [8].

Ozone therapy (OT) and Hyperbaric Oxygen Therapy (HBO) may stimulate cell proliferation and soft tissue healing reducing pain [9–12]. Laser applications at low intensity (low level laser therapy (LLLT)) have been reported in the literature for the treatment of BRONJ. Biostimulant effects of laser improve reparative process, increase inorganic matrix of bone and osteoblast mitotic index, and stimulate lymphatic and blood capillaries growth [13–16]. OT, HBO, and LLLT are in general recommended in addition to medical or surgical therapy: good clinical results are probably associated with an improvement of traditional treatments by these adjunctive therapies.

Surgical necrotic bone debridement or resection in combination with antibiotic therapy may offer long-term palliation with resolution of acute infection and pain [17]. Mobile segments of bony sequestrum and necrotic tissue should be removed extending surgery until unaffected bone is reached [18]. For diffuse BRONJ, the resection of mandible followed by reconstruction with free fibula flaps has been proposed [17–19]. In the case of large and complex surgical interventions a careful evaluation of the general conditions of each patient should be performed, including disease severity, age, and life expectancy.

The position paper of AAOMS suggested to limit surgical procedures to stage III BRONJ, but many subsequent studies reported very good results of surgery also in early stages of BRONJ.

Currently, there is no agreement with regard to the treatment of choice for stage I BRONJ and no effective unique therapy has yet been developed. The noninvasive management of these conditions is related to the prevention of the possible extension of the necrotic process, but many authors reported better results with surgical therapy than with medical treatment alone and proposed an implementation of surgical procedures, in the cases uncontrolled by local or general therapy, to limit the risk of evolution to stage III [20–23] (Table 2).

A limited surgical approach in patients not responding to noninvasive medical or adjunctive therapy (OT, HBO, and LLLT) represents a good solution. Such a treatment is rapid, poorly invasive and can be performed under local analgesia in day-surgery regimen. Here we report our experience of surgical conservative treatment of stage I in a cohort of cancer and noncancer patients under BPT with long-term followup.

## 2. Materials and Methods

This study reports the clinical outcomes of 63 patients treated for BRONJ stage I (according to Ruggiero's staging system) at the Oral Pathology and Laser-Assisted Surgery Unit of the University of Parma, Italy, between January 2004 and January 2011.

This study was approved by the Parma Hospital IRB and all participants signed an informed consent agreement.

In this retrospective analysis we included patients under BPT for malignancies or osteoporosis, with asymptomatic bone exposure in the maxillofacial region persisted for more than 8 weeks, without history of radiation therapy in the cervicofacial area.

Eligibility criteria for the retrospective analysis are shown as follows.

*Inclusion Criteria*

1. Patients under BPT for malignancies or osteoporosis with diagnosis of BRONJ in stage I.
2. Patients unresponsive to noninvasive treatments (namely, local antiseptics, antibiotic therapy, and low level laser therapy) for six months.
3. Patients considered sufficiently in health status to tolerate the surgical intervention (ASA score < 3).
4. Absence of metastasis in the region of bone exposure and the absence of deeper involvement of BRONJ.
5. Minimum followup of 6 months after surgery.

*Exclusion Criteria*

1. Presence of symptoms (pain) or clinical/radiological findings typical for different stages of BRONJ (erythema, purulent drainage, necrotic bone extending beyond the region of alveolar bone resulting in pathologic fracture, extraoral fistula, oral antral/oral nasal communication, or osteolysis extending to the inferior border of the mandible or the sinus floor).
2. Patients with history of radiation therapy in the maxillofacial region.
3. Patients immunocompromised (white blood cells < 2000 cells/mm^3^), debilitated (ASA score ≥ 3), or with serious problems of haemostasis not able to tolerate surgical intervention (platelets count < 50000 ptl/mL or INR > 3,5).
4. Patients not in agreement with specific informed consensus for surgical intervention.

Surgical interventions were performed, under local analgesia, in patients unresponsive to a period of six months of noninvasive treatments such as cycles of local or systemic antibacterial therapy combined or not to LLLT, OT, or HBO. All interventions were performed after the consultation of oncologist or physician.

For all patients all exams previously performed by their specialists (blood exams, magnetic nuclear resonance, scintigraphy, PET, and MOC) were obtained. Specific exams for BRONJ were dental X-rays, orthopantomographs, and computed tomography in order to exclude metastases in the region of bone exposure and deeper involvement of BRONJ (maxillary sinus, mandibular body and bone fractures).

The decision of BPT discontinuation before and after surgical intervention was made by the oncologist or internist.

The outcome parameters of clinical success of treatment were the absence of symptoms (pain, dysesthesia, or anaesthesia) and the presence of intact mucosa in the previous site of BRONJ without signs of infection (swelling, abscess, and fistulas) (stage 0) (Table 3) and the absence of new exposed bone near surgical area.

Data about patients, BRONJ features, and clinical outcome after surgery were summarized in tables.

*Surgical Management of Stage I BRONJ*. On the basis of BRONJ pathogenesis the aim of intervention is the complete elimination of necrotic bone followed by covering of the presumptive healthy tissue with the vascularized soft tissue of the access flap.

**Figure 1:**
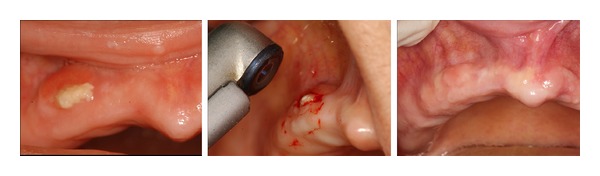
Maxillary stage I BRONJ developed in a patient who received infusions of zoledronic acid for metastasis of a breast cancer. Successfull treatment with Er:YAG laser.

**Figure 2:**
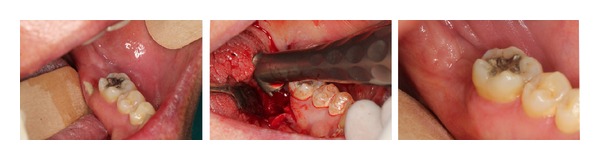
BRONJ on the left mylohyoid crest in a patient affected by multiple myeloma—complete healing achieved after Er:YAG surgery.

**Figure 3:**
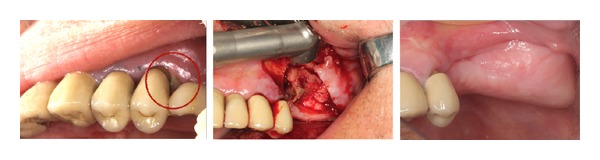
Left maxillar BRONJ stage I in a patient treated with zoledronic acid for brest cancer.

All surgical interventions were performed under local analgesia. Prophylactic antibiotics were administered for 4 days before surgery (amoxicillin and clavulanic acid 1 gr twice a day and metronidazole 500 mg twice a day) and continued postoperatively for two weeks. The surgical procedure included a mucoperiosteal flap through a linear mucoperiosteal cut surrounding bone exposure without lateral incisions to limit the risk of reduction of vascularization. The inflamed margins of the mucosa were eliminated for two millimetres to obtain a better quality tissue to cover bone surgical area. Necrotic bone was resected with surgical drills or evaporated with an erbium laser (Er:YAG laser, wave length 2940 nm, parameters: 250 mJ 20 Hz (VSP) with a fluence of 50 J/cm^2^ up to 300 mJ, 30 Hz, and fluence of 60 J/cm^2^) until the appearance of bleeding bone under sterile saline solution irrigation (Figures 1, 2, and 3).

Bone fragments and spikes were eliminated to obtain a smooth surface in order to avoid local traumatisms and to facilitate soft tissue healing over the surgical site. The surgical sites were abundantly rinsed with iodopovidone solution (10%). All surgical specimens underwent histopathological examination to confirm BRONJ diagnosis and to exclude the presence of metastasis or myeloma localization.

Wound closure was obtained by a tension-free mucosal flap sitting passively over the bone with silk suture. The sutures were removed 10 to 14 days after surgical intervention.

In the postoperative period nonsteroid anti-inflammatory drugs (NSAID), in the case of necessity, and chlorhexidine 1% gel, four times a day, were recommended.

During the post-operative follow-up each patient was visited weekly during the first month, twice a month for the two following months and once a month for the following six months. The followup was maintained every four months. Photographs were obtained before surgical interventions and during the follow-up period. The orthopantomographs, in the cases of complete mucosal healing, were obtained after six months from the surgical intervention. In cases of worsening or relapses of BRONJ new imaging exams were suddenly required.

For each patient, conforming to a protocol that satisfied the ethical standard as described by the Azienda Ospedaliero-Universitaria di Parma and University General Hospital of Valencia, Spain, we collected specific informed consensus for surgical intervention.

The algorithm of management of BRONJ in stage I is resumed as follows.

*Algorithm of Stage I BRONJ Management*

1. Diagnosis of stage I BRONJ: asymptomatic bone exposure in the maxillofacial region after 8 weeks of observation without history of radiation therapy in the cervicofacial area.
2. Photographs: at the first visit and during the follow-up period.
3. Prescription of radiographic exams: endoral RX, orthopantomographs and computed tomography.
4. Noninvasive treatment: for six months medical therapy (intermittent cycles of local or systemic antibacterial therapy) combined or not to alternative therapies (LLLT, OT, or HBO).
5. Evaluation of laboratory exams (blood exams including full blood count and hemostasis, epatic and renal function, magnetic nuclear resonance, scintigraphy, PET scan, MOC, etc.), and consultation with specialists (oncologists, physicians) required by their specialists.
6. Evaluation of evolution of disease: age, performance status and life expectancy.
7. Collection of informed consensus for surgical intervention.
8. BPT interruption (drug holiday): not in every case. The decision was made by the oncologists or internists on the basis of each single condition and necessity.
9. Prophylactic antibiotic therapy: amoxicillin and clavulanic acid 2 gr a day and metronidazole 1 gr a day starting 4 days before surgery.
10. Local analgesia: articaine 4%.
11. Mucoperiosteal envelope flap through a linear cut surrounding bone exposure without lateral discharge incisions.
12. Elimination of necrotic bone: with surgical drills or erbium laser to obtain a smooth surface of bleeding bone.
13. Irrigation of surgical site: with 10% iodopovidone solution.
14. Suture: 4 zero silk, tension free mucosal flap.
15. Removing of sutures: between 10 and 14 days after surgery.
16. Postoperative medical therapy: antibiotics for two weeks postoperatively, chlorhexidine 1% gel 4 times daily, and NSAID (if necessary).
17. Histopathological analysis of bony fragment: to confirm BRONJ and to exclude cancer diagnosis.

Followup is as follows:once a week during the first month, twice a month for the two following months, and once a month for six months. The followup will be maintained every four months.

## 3. Results

Nineteen patients were affected by multiple myeloma (MM), 29 were treated for bone metastases (BM) (48 patients, Cancer Group—CG), and 15 were taking BPs for osteoporosis (Noncancer Group—NCG). Mean BPT duration was 25.65 months for patients in CG and 90.85 months for NCG (Table 4).

According to oral subsite involved, 8 and 7 patients of CG had maxillary and mandibular involvement, respectively. In NCG, 20 patients had maxillary BRONJ while 28 had mandibular involvement. A number of patients with stage 0 disease and months of followup are shown in Table 5.

Table 6 reports the number of cases in stage I, stage II, and stage III, subclassified according site of involvement, primary disease of the patients, and treatment modality (surgical treatment, nonsurgical treatment).

Number of sites as well as percentage of complete healing are reported in Table 7.

## 4. Discussion

Marx et al. suggested in 2007 morning fasting serum c-terminal telopeptide (CTX)-guided drug holiday protocol for planning surgical procedures in patients under BPT [24]. Nowadays CTX test represents a controversial matter because it is not reliable in the cancer or rheumatoid patients under previous treatment with methotrexate, prednisone, and raloxafene because drugs and malignancy effects on bone confound the results of the test. In fact studies showed higher level of CTX in patients with bone metastasis and lower level of CTX in patients under suppressive therapies [25]. On the other hand different authors found normal rate of CTX or other bone turnover markers in BRONJ patients showing the absence of specific relationship between serum levels and severity of disease [26–29]. Some authors reported that BPs discontinuation for a variable period (one to six months) before and after interventions favoured the surgical outcome [30]. It is still unclear if long-term drug holiday can be beneficial in stabilizing sites of BRONJ or can improve the healing after surgical procedures. The discontinuation of BPT could result in a recurrence of bone pain, progression of metastases or osteolytic lesions, or increase of related skeletal events (RSE) [31, 32]. Based on the above-mentioned considerations we did not use bone metabolism markers in our case series. To plan surgical intervention we judged general health status and blood exams. Discontinuation of BPT before surgery seems not to influence the outcome in patients with stage I disease.

Some authors reported that surgery is more successful in patients with osteoporosis or multiple myeloma than in those with solid tumors. In our experience, patients treated with early surgical approach had similar percentages of healing in the 2 groups.

Wutzl et al. and Curi et al. reported that surgical procedures in patients suffering from BRONJ (also in the cases of stage I) were made under general anaesthesia. In our experience it was possible to perform interventions in day surgery under local analgesia in all cases [30, 33].

Laser can be used for conservative surgery whereby necrotic bone is vaporised, until healthy bone is reached. The erbium laser penetrates the hard tissue for 0.1 mm, providing safety guarantees and allowing precision [34]. A gradual evaporation of the necrotic bone can be performed till healthy bleeding bone is seen. The minimally invasive technique of evaporation allows the sectioned bone surfaces to be regular and can be used to create microperforations at the base for stimulating new vascularization [35]. The additional advantages of laser surgery are the bactericidal and biostimulatory actions of the laser beam with a better postoperative recovery [36].

The percentages of clinical success in BRONJ treatment reported in the literature with this technique are very high in comparison to conventional surgery [37–39]. The results in the present study confirm that the laser surgery represents a valid therapeutic option for BRONJ and enables the minimally invasive treatment of the early stages of the disease.

## 5. Conclusions

When making the decision to perform surgical procedures for the treatment of BRONJ, the deal between benefit and potential risks according to clinical circumstances of each patient should be considered. Surgical operations for advanced stages of BRONJ are invasive and extensive and must be performed under general anaesthesia. Only few patients may undergo this type of surgery. On the other hand a minimal and faster intervention under local analgesia is useful also for aged and immunocompromised patients. Less invasive surgery may determine a complete mucosal healing containing the microbial infection and the risk of spread of the disease.

Our result confirms that treatment of patients affected by minimal bone exposition, (stage I of BRONJ), through conservative surgical strategies, possibly with laser, may determine a higher control of lesions in the long term.

## Figures and Tables

**Table 1:** Clinical classification of BRONJ by Ruggiero et al. [3] (2009).

BRONJ stage	Description	Treatment strategies
At risk category	No apparent necrotic bone in patients who have been treated with either oral or IV bisphosphonates	No treatment Patients education

Stage 0	No clinical evidence of necrotic bone, but nonspecific clinical findings and symptoms	Systemic therapies including pain medications and antibiotics

Stage I	No symptomatic lesions with bone exposure in absence of signs of infection	Topical antiseptic therapy Follow-up

Stage II	Bone exposure with pain, infection, and swelling in the area of lesion	Oral antibiotics—antibacterial mouth rinse-pain control Superficial debridement to relieve soft tissues irritation

Stage III	Bone exposure, pain, inflammation, maxillary sinus involvement, cutaneous fistulas, and pathological fractures	Antibacterial mouth rinse Antibiotic therapy and pain control Surgical debridement and resection for longer term palliation of infection and pain

Modified from [3].

**Table 2:** Clinical staging and management strategies by Bagan et al. [22] (2009).

Stage I	Exposed bone necrosis or small oral ulceration without exposed bone necrosis, but without symptoms
Stage II	Exposed bone necrosis or a small oral fistula without exposed bone necrosis, but with symptoms controlled with medical treatment
Stage II	Exposed bone necrosis or a small oral fistula without exposed bone necrosis, but with symptoms not controlled with medical treatment
Stage III	Jaw fractures, skin fistula, and osteolysis extending to the inferior border

Bagan and coll. staging system for BRONJ (modified from [22]).

**Table 3:** Staging system of “clinical success” in the BRONJ management by Vescovi et al. [13] (2006).

A	Stage 0	Complete mucosal healing, no symptoms, and no infection signs

B	Stage I	Presence of bone exposure, regression of infection signs, and regression of symptoms
	Stage II	Presence of bone exposure with pain, infection and swelling in the lesion area, disappearance of cutaneous fistulas, maxillary sinus infection, and fracture reparation
	Stage III	Presence of bone exposure, pain, inflammation, secondary infections, cutaneous fistulas, and pathological fractures

Level A and level B should be maintained for at least 6 months after therapy.

**Table 4:** Patients with stage I BRONJ evaluated in the present study.

Disease	Patients	BPT duration (months ± SD)	Range (months)
Multiple myeloma	19	25.65 ± 20.3	3–72
Bone metastasis	29		
Osteoporosis	15	90.85 ± 40.4	24–144

**Table 5:** Sites of occurrence of BRONJ.

Disease	Sites	Patients	Stage 0	Follow-up range (months)
Nononcological patients	Max	8	4	6–29
	Mand	7	3	

Oncological patients	Max	20	10	6–50
	Mand	28	12	

**Table 6:** Outcome of surgical and nonsurgical treatments.

	Sites	Max	Mand	CA	MM	OP	BPT INT	BPT CONT
Stage I								
Nonsurgical treatment								
Complete healing	4	2	2	4	—	—	2	2
No healing	32	18	14	14	10	8	15	17
Surgical treatment								
Complete healing	25	12	13	10	8	7	12	13
No healing	2	1	1	1	1	—	1	1

Stage II								
Nonsurgical treatment								
Complete healing	13	3	10	3	3	7	6	7
No healing	39	14	25	15	15	9	25	14
Surgical treatment								
Complete healing	50	14	36	22	19	9	30	20
No healing	16	3	13	8	8	—	8	8

Stage III								
Nonsurgical treatment								
Complete healing	0	—	—	—	—	—	—	—
No healing	7	2	5	3	2	2	5	2
Surgical treatment								
Complete healing	3	3	—	—	—	3	2	1
No healing	1	—	1	—	1	—	—	1

Number of sites as well as percentage of complete healing are reported in Table 7.

**Table 7:** Number and percentage of healed sites after BRONJ treatment.

	Sites	%
Stage I		
Nonsurgical treatment		
Complete healing	4	11.2
No healing	32	88.8
Surgical treatment		
Complete healing	25	92.6
No healing	2	7.4

Stage II		
Nonsurgical treatment		
Complete healing	13	25
No healing	39	75
Surgical treatment		
Complete healing	50	75.5
No healing	16	24.25

Stage III		
Nonsurgical treatment		
Complete healing	0	0
No healing	7	100
Surgical treatment		
Complete healing	3	75
